# Carbon and Water Use Efficiencies: A Comparative Analysis of Ten Terrestrial Ecosystem Models under Changing Climate

**DOI:** 10.1038/s41598-019-50808-7

**Published:** 2019-10-11

**Authors:** Bassil El Masri, Christopher Schwalm, Deborah N. Huntzinger, Jiafu Mao, Xiaoying Shi, Changhui Peng, Joshua B. Fisher, Atul K. Jain, Hanqin Tian, Benjamin Poulter, Anna M. Michalak

**Affiliations:** 1 Department of Earth and Environmental Sciences, Murray State University, Murray, KY 42071 USA; 2 Woods Hole Research Center, Falmouth, MA 02540 USA; 3 School of Earth Sciences and Environmental Sustainability, Northern Arizona University, Flagstaff, AZ 86011 USA; 4 Environmental Sciences Division and Climate Change Science Institute, Oak Ridge National Laboratory, Oak Ridge, 37831 TN USA; 5 Department of Biological Sciences, University of Quebec at Montreal, Montréal, QC H3C 3J7 Canada; 6 Jet Propulsion Laboratory, California Institute of Technology, Pasadena, CA 91109 USA; 7 Department of Atmospheric Sciences, University of Illinois at Urbana-Champaign, Urbana, IL 61801 USA; 8 International Center for Climate and Global Change Research, School of Forestry and Wildlife Sciences, Auburn University, Auburn, AL 36849 USA; 9 NASA Goddard Space Flight Center, Greenbelt, MD 20771 USA; 10 Department of Global Ecology, Carnegie Institution for Science, Stanford, CA 94305 USA

**Keywords:** Ecological modelling, Ecophysiology

## Abstract

Terrestrial ecosystems carbon and water cycles are tightly coupled through photosynthesis and evapotranspiration processes. The ratios of carbon stored to carbon uptake and water loss to carbon gain are key ecophysiological indicators essential to assess the magnitude and response of the terrestrial plant to the changing climate. Here, we use estimates from 10 terrestrial ecosystem models to quantify the impacts of climate, atmospheric CO_2_ concentration, and nitrogen (N) deposition on water use efficiency (WUE), and carbon use efficiency (CUE). We find that across models, WUE increases over the 20^th^ Century particularly due to CO_2_ fertilization and N deposition and compares favorably to experimental studies. Also, the results show a decrease in WUE with climate for the last 3 decades, in contrasts with up-scaled flux observations that demonstrate a constant WUE. Modeled WUE responds minimally to climate with modeled CUE exhibiting no clear trend across space and time. The divergence between simulated and observationally-constrained WUE and CUE is driven by modeled NPP and autotrophic respiration, nitrogen cycle, carbon allocation, and soil moisture dynamics in current ecosystem models. We suggest that carbon-modeling community needs to reexamine stomatal conductance schemes and the soil-vegetation interactions for more robust modeling of carbon and water cycles.

## Introduction

Terrestrial ecosystems assimilate atmospheric CO_2_ through photosynthesis, where carbon sequestered is accompanied by loss of water to the atmosphere as regulated by leaf stomata. The rate of net carbon uptake (net primary production, NPP) to gross carbon uptake (gross primary production, GPP) is referred to as carbon use efficiency (CUE). CUE plays an important role in the terrestrial ecosystem carbon balance^1^ and determines the amount of carbon allocated to biomass. An additional metric of plant resource economy quantifies the rate of carbon uptake per unit of water loss. This water use efficiency (WUE) is a key physiological parameter linking carbon and water cycles. WUE quantifies the amount of water that terrestrial ecosystems use relative to carbon gained^2,3^. CUE and WUE are important indicators of plants ability to adapt to changing environmental conditions, such as precipitation and temperature. Understanding changes in CUE and WUE is critical to quantify the response of terrestrial ecosystems to the changing climate^4–7^.

Variability in CUE and WUE efficiencies is a reflection of the ecosystem dynamics in a changing environment. Low CUE can be an indication of high respiratory cost associated with warmer temperature, longer growing season, and nutrient deficiencies^1,8^. High CUE can be an indication of adaptation to harsh environmental conditions such as cold temperature and insufficient precipitation^9^. High WUE can indicate low stomatal conductance resulting in reduced transpiration^10^, which is the case of plants growing in arid conditions. A low WUE can indicate sufficient soil moisture or precipitation and enhanced tree growth. Under a projected warming climate (hotter and drier environmental conditions., CUE is expected to decrease as respiratory cost increases^11^, while WUE is expected to increase due to reductions in stomatal conductance^12^, resulting in limited tree growth^13–16^.

The leaf ecophysiological properties indicate that CUE and WUE should increase due to increasing atmospheric CO_2_- known as “the CO_2_ fertilization effect”. CO_2_ enrichment experiments^3,17,18^, ecosystem models, isotope analysis^19–21^, and WUE model (WUE estimated independently from GPP and evapotranspiration)^22^, support an increase in plant WUE as atmospheric CO_2_ concentration increases. However, the response of CUE to changes in atmospheric CO_2_ levels is largely unknown leading to a debate about the role of nutrients limitations^10,19^. This is not surprising given the difficulties in estimating CUE at sites and the difficulties in extrapolating the limited CUE estimates from a local to a global scale. Understanding how the changing climate induces changes in WUE and CUE is of critical importance to improve our abilities to accurately predict any future ecosystem changes.

Here, we analyze trends in global CUE and WUE using an ensemble of ten ecosystem models (see Methods) from the Multi-scale Synthesis and the Terrestrial Model Intercomparison Project (MsTMIP)^23^. In order to achieve this, we use an “assumption-centered“ approach^24^ to identify key model processes related to estimating CUE and WUE and to evaluate the assumptions in these processes against published studies and the globally upscaled flux tower observations^25^. We focus on quantifying the long-term impacts of atmospheric CO_2_, climate change, and nitrogen deposition as well as their combined effects on annual modeled CUE and WUE. We address a variety of factors that can influence the models’ trend, including the potential role of autotrophic respiration and carbon allocation. Furthermore, the intermodel analysis focuses on determining the dominant environmental drivers of the modeled CUE and WUE. This allows for models’ structure to be assessed, while providing intercomparison of the modeled CUE and WUE driver’s spatial and temporal variability.

## Results and Discussion

We find that models disagree in terms of the magnitude and trend of WUE and CUE, which reflects the differences in model structure and parameterization. The trend of the models’ WUE (positive trend) and CUE (some models showed a positive trend, while others showed a negative trend) is significant (Mann-Kendall p < 0.05) for CO_2_ fertilization and N deposition scenarios and shows contrasting results for the climate scenario with about half of the models showing no significant trend with time (Tables S1 and S2).

**Figure 1:**
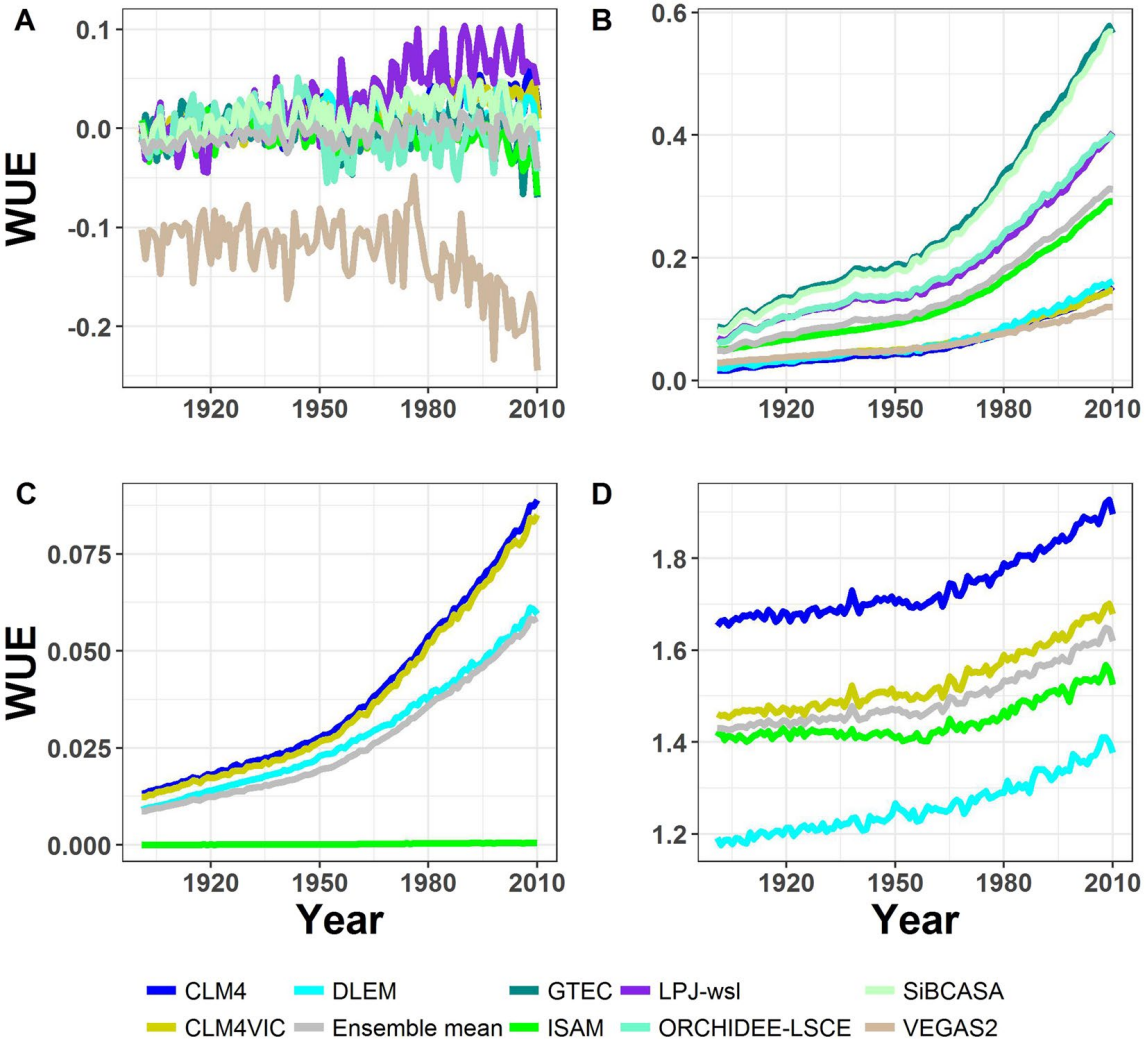
Effect of environmental variables on the annual changes in WUE from 1901–2010. (**a**) climate effect on models WUE calculated as the difference between SG1-RG1 simulations, (**b**) CO_2_ fertilization effect on models WUE calculated as the difference between SG3-SG2, (**c**) N deposition effect on models WUE calculated as the difference between BG1-SG3, and (**d**) the interactions of climate, CO_2_ fertilization, and N deposition (BG1 simulation) effects on models WUE calculated as the net change in WUE relative to year 1901 (See Supplementary Information).

We compare the effects of the changing climate on modeled WUE (Fig. 1a) to FLUXNET-MTE for the period of 1982–2008 (Fig. S1). Only four models show a significant decreasing trend (Mann-Kendall p < 0.05) with a drop in carbon sequestered per unit of water lost (increase in leaf transpiration; see Methods) ranging between −1.4% to −25.6% for WUE, while the remaining models show no trend (Table S1). Meanwhile, no significant trend (p = 0.6) in FLUXNET-MTE WUE for the same period is detected (Fig. S1; Table S1). Discrepancies between models and FLUXNET-MTE is due to differences in the representation of soil hydrology and the interconnectedness of stomatal conductance and carbon assimilation between models. For example, CLM4 shows similar WUE values to FLUXNET-MTE with a mean absolute error of 0.04 gC/kgH_2_O, unlike CLM4VIC that underestimated FLUXNET-MTE WUE with a mean absolute error of 0.18 gC/kgH_2_O (Fig. S1). Implementation of VIC hydrology in CLM4 resulted in an increase in soil moisture and a decrease in WUE.

**Figure 2:**
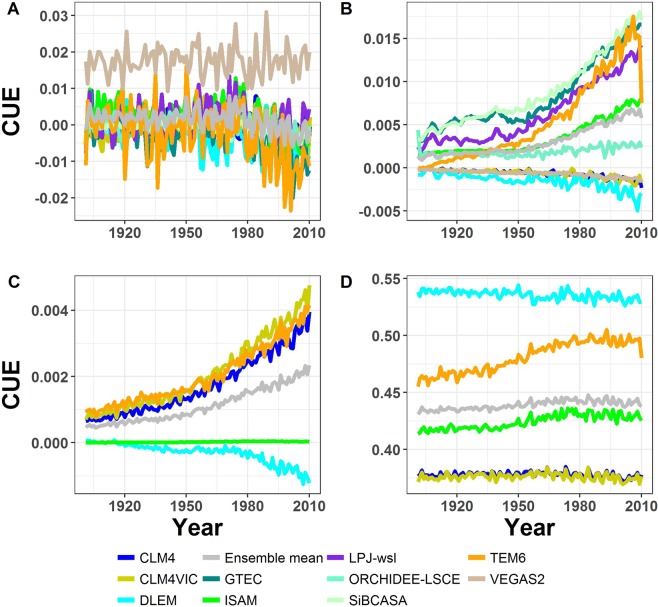
Effect of environmental variables on the annual changes in WUE from 1901–2010. (**a**) climate effect on models CUE calculated as the difference between SG1-RG1 simulations, (**b**) CO_2_ fertilization effect on models CUE calculated as the difference between SG3-SG2, (**c**) N deposition effect on models CUE calculated as the difference between BG1-SG3, and (**d**) the interactions of climate, CO_2_ fertilization, and N deposition (BG1 simulation) effects on models CUE calculated as the net change in CUE relative to year 1901 (See Supplementary Information). The sudden drop in TEM6 CUE in (**b**,**d**) is due to a decrease in TEM6 NPP and GPP for year 2010.

The differences we find in modeled CUE and WUE are due to models’ representation of the interactions between climate, atmospheric CO_2_ concentration, and N-deposition. Whether a model considers C-N coupling appears to influence WUE and CUE sensitivities to an increase in atmospheric CO_2_. On average, C-N models show a weaker WUE response to CO_2_ fertilization than C only models (Fig. 1b; Table S4). Models, with the exception of VEGAS2.1, predicted an increase in WUE due to CO_2_ fertilization (Table S4) consistent with observational studies that estimated an increase in WUE ranging from 8% to 35% over the 20^th^ century^10,12,15,17,26,27^. This suggests a decrease in the models stomatal conductance with CO_2_ fertilization similar to observations^15,20,26,28^. Three of the C-N models (CLM4, CLM4VIC, and DLEM) also show a negative CUE response to CO_2_ fertilization (Fig. 2b; Table S2). Results show for models with positive CUE trend (Fig. 2b), such as ISAM, LPJ and ORCHIDEE, the percentage increase in NPP is considerably larger than the percentage increase in GPP (Table S5). Whereas, models that show negative CUE trend (Fig. 2b), such as CLM and CLMVIC, the percentage increases in NPP and GPP are the same or GPP percentage increase is greater than that of NPP (Table S5). This drastic response in models CUE is attributed in part to higher autotrophic respiration rates relative to NPP. In general, models’ autotrophic respiration structure is almost identical, but drivers of autotrophic respiration, such as carbon allocation scheme and tissue turnover vary between models. Models (e.g. CLM4 and CLMVIC) that are parameterized to favor increased tree growth (mainly wood biomass) risk increasing plant autotrophic respiration and decreasing CUE with CO_2_ fertilization (Table S3). In addition, the discrepancy between the models’ estimated WUE with climate change (Fig. 1a) can be due to either an increase in stomatal conductance or in photorespiration that leads to a decrease in GPP. Further experimentation is needed to modify model processes (e.g. carbon allocation and autotrophic respiration) that lead to near constant CUE with time to preserve the inter-annual and seasonal variability in CUE as observed in field studies^29–31^ and to accurately capture stomatal conductance response to changing environmental conditions to improve models’ prediction of the carbon and water cycles.

The differences in model response to N deposition can be traced to GPP-N relationships. Models showed a slight increase in WUE with N deposition between years 1901 and 2010 (Mann-Kendall p < 0.05) with a % change ranging from 0.07% to 0.8% (Table S1). This is similar to recent long-term studies that showed a slight increase in WUE (intrinsic WUE) with N deposition at site and regional scales^32–34^. On average models GPP increased by about 4% more than models evapotranspiration (0.5%), resulting in the apparent increase in models WUE with N deposition (Fig. 1c). In addition, CLM potential GPP is limited by N requirements for new tissue growth^35,36^ while in ISAM GPP is limited by N down regulation of V_cmax_^37^. Thus, additional N resulted in a slight increase in NPP in CLM4 and CLM4VIC due to an increase in LAI (Fig. S2), while ISAM is less sensitive to N deposition. Moreover, ISAM CUE and WUE exhibit little to no effect by N deposition, suggesting that the carbon allocation scheme is not sensitive to N deposition and that the structural growth-N deposition relationship needs to be modified to improve the response of ISAM to N deposition (Fig. 2c). Nevertheless, all models show minimal impacts of N deposition on CUE. Thus, modifications of N deposition interactions with leaf N and V_cmax_ are recommended to improve model performance.

**Figure 3:**
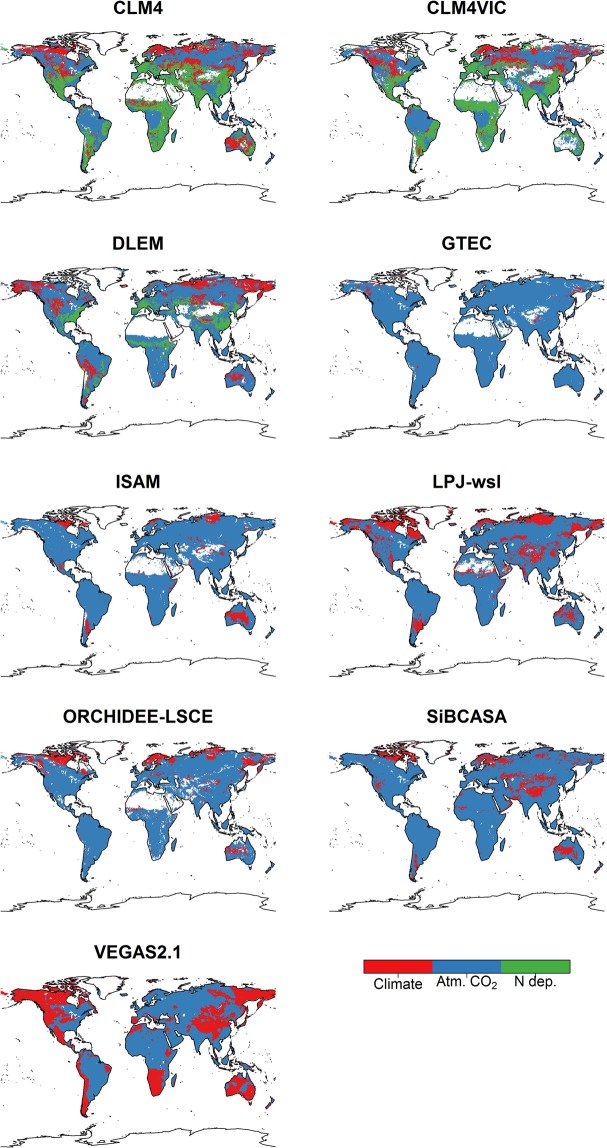
Spatial variability in the dominant environmental driver (Climate, CO_2_ fertilization and N deposition) on the average models WUE for 1982–2008. The averaged modeled WUE is for BG1 scenario (C-N models: CLM4, CLM4VIC, DLEM, and ISAM) and SG3 scenario (C only models) (See Supplementary Information).

**Figure 4:**
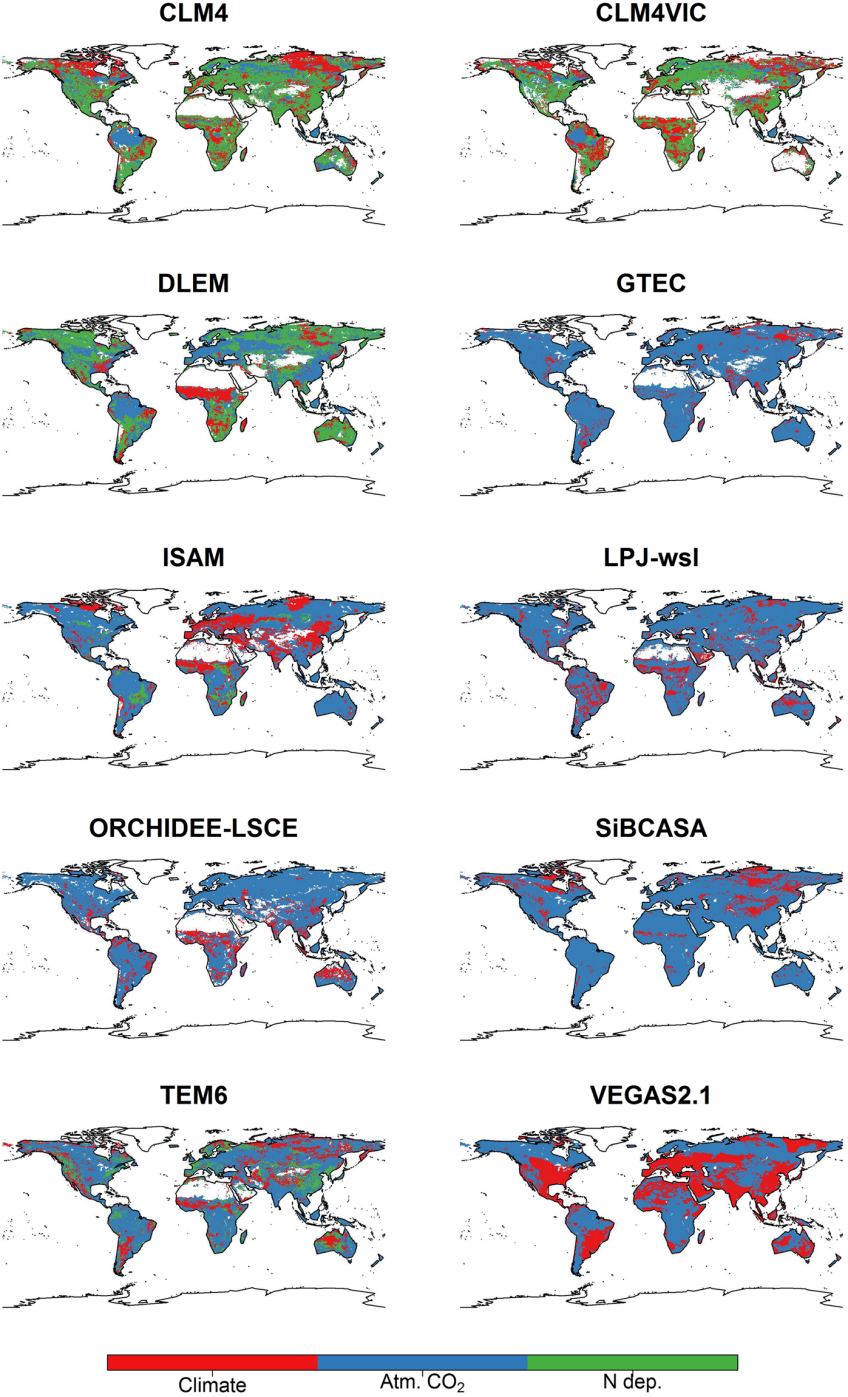
Spatial variability in the dominant environmental driver (Climate, CO_2_ fertilization and N deposition) on the average models CUE for 1982–2008. The averaged modeled CUE is for BG1 scenario (C-N models: CLM4, CLM4VIC, DLEM, ISAM, and TEM6) and SG3 scenario (C only models) (See Supplementary Information).

There is an agreement on the dominant drivers of WUE and CUE between models (Figs 3, 4 and S3). CO_2_ fertilization is the main driver for CUE and WUE changes in most models (Table S6). Our conclusion is similar to other studies that found models net carbon uptake is CO_2_ dependent and highly variable between LSMs^27,38^. However, N deposition is the main driver for two models (CLM4 and CLM4VIC) and climate is the main driver for one model (VEGAS2.1) (Figs 3, 4 and S3). The dominance of N deposition as the environmental driver of CLM4 and CLM4VIC can be an indication of the models strong N limitation because CO_2_ fertilization of GPP is calculated independent of N limitations and then downregulated if N uptake is insufficient to support GPP^39^. The dominance of CO_2_ fertilization as the main environmental drivers to WUE, CUE, GPP, and NPP is more consistent among C only models than C-N models (Figs 3, 4 and S4–6). It is possible that the difference between C-N and C only models is due to different model parametrizations and structure^23^, such as stomatal conductance scheme and whether stomatal conductance is parametrized for the whole canopy or sunlit/shaded leaves.

Models disagree on the spatial importance of all environmental drivers^19^ with low agreement in the temperate and the boreal-artic zones and high agreement in the tropical zone (Figs 3 and 4). In the arctic and boreal regions, models’ CUE and WUE are driven by atmospheric CO_2_ levels and, to a lesser extent, by the changing climate (Figs 3 and 4). This contrast studies showing that climate is the dominant contributor to the increase in intrinsic WUE in boreal forest in the last five decades^21,40^. Also, there is a significant disagreement for the dominance environmental drivers for topical Asia with three models (LPJ-wsl, ORCHIDEE- LSCE, and VEGAS2.1) showing climate as the important driver, unlike the rest of the models (Fig. 3). Nevertheless, we find that the spatial dominance of all environmental drivers of models CUE and WUE follows a similar spatial pattern to that of GPP and NPP (Figs S4 and S5). Whereas, climate is the dominate environmental driver of ET for almost all models (Fig. S6), but is not as prominent driver for models’ WUE. This is related to the fact that the % change in models’ GPP is much larger than that of ET (Table S5). The small effects of CO_2_ fertilization on ET is not due to differences in evapotranspiration scheme between models, but rather due to models assumptions that can lead to lower CO_2_ effects on ET (e.g. decoupling of assimilation and transpiration and whether soil moisture stress modifies assimilation and stomatal conductance). Models high sensitivity to CO_2_ fertilization and low sensitivity to climate (Figs 3, 4 and S2) is a result of indirect tuning of models sensitivities to environmental drivers^38^. We argue that autotrophic respiration and GPP schemes needs to be modified to improve their sensitivity to temperature and CO_2_ fertilization for better projection of the carbon sink magnitude under changing environmental conditions.

We find that latitudinal variability in soil moisture and temperature (Figs S7 and S8) is not directly related to that of CUE and WUE (Figs S9 and S10). For instance, CLM4 and GTEC soil moisture show a near constant response to climate and CO_2_ fertilization (Fig. S7a,b), unlike CLM4 and GTEC CUE and WUE (Figs S9 and S10). Even models with different soil hydrology (CLM4 and CLM4VIC) show similar CUE and WUE sensitives to environmental drivers (Figs S9 and S10). The low sensitivity of above ground processes to soil moisture and temperature suggest either inaccurate models assumptions or decoupling between soil moisture and CUE and WUE. We note that models soil temperature respond to climate driver (Fig. S8a) is consistent with the observed increase in soil temperature across the globe^41–45^. The decrease in models’ temperature (except LPJ-wsl and GTEC) due to CO_2_ fertilization (Fig. S8b) can reduce the effect of CO_2_ fertilization on CUE and WUE^46^, but such an affect is not apparent in models’ CUE and WUE (Figs S9 and S10). Nevertheless, accurate representation of soil hydrology and better assumptions about GPP-ET interactions^47^ are keys to improve models’ prediction of environmental drivers of terrestrial ecosystems.

Future studies need to investigate the soil-vegetation interactions and assumptions in ecosystem models and how such interactions/assumptions impact vegetation CUE and WUE. For instance, what is the role of soil moisture or water stress on model WUE? More information about model assumptions is needed to answer this question. However, it does seem that CO_2_ fertilization causes stomatal conductance to increase regardless of soil water availability. Moreover, carbon isotope studies suggest a constant ratio of intercellular CO_2_ (ci) and atmospheric CO_2_ (ca) [ci/ca ratio] with increasing atmospheric CO_2_ concentration^15,18,48^ and depending on temperature, vapor pressure deficit and elevation. Does this suggest that models with decreasing WUE due to CO_2_ fertilization are not maintaining a near constant ci/ca ratio? We hypothesize that this is the case in ecosystem models, but we know very little to nothing about the models ci/ca ratio to verify our hypothesis because ci/ca ratio is not a typical ecosystem models output.

Detailed analysis of ecosystem models CUE and WUE provides necessary information about model assumptions and parameterizations. On average, we find that models predict WUE more accurately and show contrasting results for CUE, particularly for climate change and nitrogen deposition scenarios. The uncertainties and errors in CUE can be mostly attributed to NPP and autotrophic respiration, nitrogen cycle, and carbon allocation schemes in current ecosystem models. Our analysis also suggests that ecosystem models should consider the acclimation of ecosystems to rise in temperature in order to increase the sensitivity of CUE to climate. Future model intercomparison studies^24,47,48^ should move beyond model results comparison and should seek to understand and identify the key processes that will lead to better model performance and will reduce uncertainties in the future projections. Key processes can be identified by: (1) Synthesis of existing data (e.g. turnover rates, allocation, soil temperature and moisture) to constrain and reduce models uncertainties; and (2) Comprehensive analysis of models behavior and validation at finer temporal scales (daily and diurnal cycle) for important variables (carbon and water fluxes as well as soil temperature and moisture) at multiple sites (e.g. FLUXNET sites) to pinpoint exact processes and parameters that require modification. We call on new intercomparison studies focus not only on vegetation variables (e.g. GPP, NPP, etc.), but also on soil physical and chemical properties (e.g. soil moisture and temperature) because it should not be expected that models would accurately predict the carbon and water cycles unless water stress and soil moisture are accurately captured.

## Methods

### Model ensemble

The models used here are part of the Multi-Scale synthesis and Terrestrial Model Intercomparison Project (MsTMIP). To eliminate any errors that could arise between models due to the use of different environmental drivers, standardized forcing data such as climate, land cover, atmospheric CO_2_ concentration, and N deposition, were provided to the modelers. Models simulations are for the time period spanning 1901–2010 using a standard spin-up and simulation protocols^39,49^. The models are run in an offline simulation using the forcing data provided. Each simulation protocol is designed to test the sensitivity of one of the above-mentioned forcing data and the impact of each driver is calculated through simulation differencing as mentioned previously (Table S7). We only use the models that outputted GPP, NPP, autotrophic respiration, and evapotranspiration for all the simulations (Table S8). We note that evapotranspiration data are lacking for TEM6 and it is included only in the analysis of CUE.

The climate impact on modeled CUE and WUE is calculated as the difference between fixed and variable climate simulations (SG1-RG1); atmospheric CO_2_ concentration impacts on modeled CUE and WUE are calculated as the difference between fixed and variable atmospheric CO_2_ concentration simulations (SG3-SG2); and N-deposition impact on modeled CUE and WUE is calculated as the difference between fixed and variable N deposition simulations (BG1-SG3). The combined effects of climate and increases in atmospheric CO_2_ concentration and N-deposition are assessed by analyzing BG1 simulation. The models WUE for the SG1 simulation are compared to FLUXNET-MTE for years 1982–2008. We calculated the net change in WUE and CUE for the BG1 simulations as the difference between yearly WUE or CUE estimates and WUE or CUE estimates for year 1901 (net WUE[n] or CUE[n] = WUE[n] or CUE [n] – WUE[1901] or CUE[1901], where n is year).

### Statistical analysis

Mann-Kendall nonparametric test is applied to statistically detect any trend (increasing or decreasing) in WUE and CUE over time^50,51^. We use the trend free pre-whitening approach^52^ Mann-Kendall test to remove any correlation in the time series data as it has been shown to outperform the pre-whitening approach and reduce the errors in trend detection^52,53^. The null hypothesis (H_0_) of this test is no trend, and the alternative hypothesis (H_A_) that there is a trend. Mann-Kendall test ranges from −1 to 1 with values close to zero indicating no trend. Also, we use the Sen’s slope nonparametric methods to estimate the slope of the trend. Positive values of Sen’s slope indicate an increasing trend and negative values indicate a decreasing trend.

### Calculation of magnitude change as percentage of mean

We calculate percentage change as the median slope (Sen’s slope for WUE or CUE) multiplied by the period length (n) divided by the mean^54^ as:

$$Precentage\,change\,( \% )=\frac{Sen^{\prime} \,slope\times n}{mean}\times 100$$

Calculation of N deposition effect on LAI: We calculated LAI for CLM4 and CLM4VIC because they provided LAI for the BG1 simulations (ISAM LAI for this version of MsTMIP is satellite-based and not dynamically estimated, thus it is excluded from this analysis). We limit the analysis for areas north of 25° latitude since these are the areas considered N limited. We extract LAI data for months of April to October and average the LAI for each of these months to produce a time series of LAI data for years 1901 until 2010.

### Calculation of percentage changes in model data

We calculate the percentage increase in NPP, GPP, and autotrophic respiration for any simulation as the difference between 2010 and 1901 estimates. We calculate the percentage contribution of climate, CO_2_ fertilization, and N deposition to the models BG1 CUE and WUE estimates as the ratio between the average difference in simulations (e.g. SG1 - RG1) and average BG1 WUE and CUE for C-N models and SG3 WUE and CUE for C only models for year 1901–2010.

## Supplementary information

Supplementary Information

## Data Availability

The output of MsTMIP models are available from https://daac.ornl.gov/cgi-bin/dsviewer.pl?ds_id=1225.
