# Supplementary material for: Carbon and Water Use Efficiencies: A Comparative Analysis of Ten Terrestrial Ecosystem Models under Changing Climate
Source: Sci Rep. 2019 Oct 11;9:14680. doi: 10.1038/s41598-019-50808-7 (PMC6789101; doi:10.1038/s41598-019-50808-7)
Supplement: Supplementary file 1 — Supplementary Information [file 41598_2019_50808_MOESM1_ESM.docx]

**Supplementary Information for:**

**Carbon and Water Use Efficiencies: A Comparative Analysis of Ten Terrestrial Ecosystem Models under Changing Climate**

Bassil El Masri^1*^, Christopher Schwalm^2,3^, Deborah N. Huntzinger^3^, Jiafu Mao^4^, Xiaoying Shi^4^, Changhui Peng^5^, Joshua B. Fisher^6^, Atul Jain^7^, Hanqin Tian^8^, Benjamin Poulter^9^ and Anna M. Michalak^10^

^1^ Department of Earth and Environmental Sciences, Murray State University, Murray, KY 42071, USA

^2^ Woods Hole Research Center, Falmouth, MA, 02540, USA

^3^ Center for Ecosystem Science and Society, Northern Arizona University, Flagstaff, AZ 86011, USA

^4^ Environmental Sciences Division and Climate Change Science Institute, Oak Ridge National Laboratory, Oak Ridge, TN, USA

^5^ Department of Biological Sciences, University of Quebec at Montreal, Montréal, QC H3C 3J7, Canada

^6^ Jet Propulsion Laboratory, California Institute of Technology, 4800 Oak Grove Dr., Pasadena, CA, 91109, USA

^7^ Department of Atmospheric Sciences, University of Illinois, Urbana, IL, 61801, USA

^8^ International Center for Climate and Global Change Research, School of Forestry and Wildlife Sciences, Auburn University, 602 Duncan Drive, Auburn, AL 36849, USA.

^9^ NASA Goddard Space Flight Center, Greenbelt, MD, 20771, USA

^10^ School of Earth and Environmental Studies, Stanford University, CA, 94305, USA

^*^ Correspondence to: Bassil El Masri, belmasri@murraystate.edu

**CUE and WUE:**

To calculate global annual CUE and WUE, monthly global average GPP, NPP, and ET for each of MsTMIP models were calculated as the mean of all the land grid cells. Then, annual GPP_ann_, NPP_ann_, and ET_ann_ were estimated as the sum of the monthly GPP, NPP, and ET, respectively. Annual CUE and WUE were calculated for each model as:

$$CUE= \frac{{NPP}_{ann}}{{GPP}_{ann}} S1$$

$$WUE= \frac{{GPP}_{ann}}{{ET}_{ann}} S2$$

Equations S1 and S2 were used to estimate CUE and WUE for all MsTMIP simulations (see table S1). To calculate average global CUE and WUE, monthly GPP, NPP, and ET were summed for each grid cell to produce annual gridded GPP_ann_gr_, NPP_ann_gr_, and ET_ann_gr_ for each model for years 1982-2008. For each of the models, mean CUE and WUE for years 1982-2008 were calculated as:

$$CUE= \frac{mean\left( {\sum_{1}^{n} NPP}_{ann\_gr} \right)}{mean\left( {\sum_{1}^{n} GPP}_{ann\_gr} \right)} S3$$

$$WUE= \frac{mean\left( {\sum_{1}^{n} GPP}_{ann\_gr} \right)}{mean\left( {\sum_{1}^{n} ET}_{ann\_gr} \right)} S4$$

where n is year. Equations S3 and S4 were applied per grid cell for each of the MsTMIP simulations (see table S1).

Models’ CUE and WUE exhibit large divergence in their baseline estimates for year 1901. We removed the large divergence in models estimated CUE and WUE for the BG1 simulation to analyze only the net change in models’ CUE and WUE estimates and the trend with time. This was accomplished by subtracting models CUE 1901 estimate from the models CUE for each of the years from 1901 until 2010 as:

$${CUE\_net}_{y}= {CUE}_{y}-{CUE}_{1901} S5$$

where y is year. Equation S4 was applied to modeled WUE.

**Environmental drivers:**

To spatially calculate the dominant environmental drivers (climate, atmospheric CO_2_ concentration, and nitrogen deposition) on CUE and WUE, simulation differencing was performed. We calculated the percentage change in models’ CUE and WUE due to the impacts of climate (CUE_cl_, WUE_cl_), atmospheric CO_2_ concentration (CUE_co_, WUE_co_), and nitrogen deposition (CUE_nd_, WUE_nd_) in reference to the BG1 simulation as the following:

$${CUE\_perc}_{cl,co,nd}\left( \% \right)= \frac{{CUE}_{cl, co, nd}}{{CUE}_{BG1}}\times100 S6$$

$${WUE\_perc}_{cl, co,nd}\left( \% \right)= \frac{{WUE}_{cl,co,nd}}{{WUE}_{BG1}}\times100 S7$$

Finally, we determine the dominant driver for each model as the following:

$$Dominant driver=\max\left( {CUE_{per}}_{cl},{CUE_{per}}_{CO},{CUE_{per}}_{nd} \right) S8$$

Equation S8 was performed per pixel and models’ WUE as well.

**Soil Moisture and Temperature**

We calculated soil temperature and moisture as the average of the top 6 soil layers (~50 cm soil depth) for models that provided soil temperature and moisture data. We note that models that only CLM4, CLM4VIC, and SiBCASA provided soil temperature data for different soil layers, while GTEC and LPJ-wsl provided average layered soil temperature. Soil moisture was available per soil layer for all the above mentioned models, except LPJ-wsl where only layer # 1 was used as the second soil layer has no data. We restrained our analysis for 1982-2008 period to be consistent with the same time period used to estimate the dominant environmental drivers.

**FLUXNET-MTE**

FLUXNET-MTE is a global gridded carbon and water fluxes at 0.5^o^ ⨯ 0.5^o^ spatial resolution. FLUXNET-MTE data are derived from empirically up-scaling eddy covariance flux tower observations using the model tree ensemble approach [Jung et al., 2011]. FLUXNET-MTE data are available monthly from 1982-2008 and can be accessed from <https://www.bgc-jena.mpg.de/bgi/index.php/Services/Overview>. To compare the FLUXNET-MTE WUE to the models WUE, we calculated FLUXNET-MTE GPP as the difference between total respiration (Re) and net ecosystem exchange (NEE) and estimated WUE following equation S2. Then, we monthly averaged the FLUXNET-MTE WUE estimates over 1982-2008 for the gridded analysis.

**Supplementary Tables**

Table S1. Summary of statistical analysis for models WUE for 1901-2008, and SG1 (climate) and FLUXNET-MTE WUE for 1982-2008. % change is calculated based on Sen’s slope and mean WUE (See supplementary information). Mann-Kendall is estimated for simulation differencing except for BG1. Thus, only models with submitted simulations RG1 through BG1 (models with coupled C-N cycle) and RG1 through SG3 (Carbon cycle only models) are included. Models with coupled carbon-nitrogen cycle are denoted in bold.

|  | **Mann-Kendall** | **p-value** | **% change** |
| --- | --- | --- | --- |
| **BG1** | | | |
| **CLM4** | 0.41 | < 0.001 | 12.64 |
| **CLM4VIC** | 0.46 | < 0.001 | 14.33 |
| **DLEM** | 0.59 | < 0.001 | 17.45 |
| **ISAM** | 0.24 | < 0.001 | 7.61 |
| **N-deposition** | | | |
| **CLM4** | 0.76 | < 0.001 | 0.39 |
| **CLM4VIC** | 0.69 | < 0.001 | 0.80 |
| **DLEM** | 0.73 | < 0.001 | 0.36 |
| **ISAM** | 0.48 | < 0.001 | 0.07 |
| **CO_2_** | | | |
| **CLM4** | 0.62 | < 0.001 | 1.45 |
| **CLM4VIC** | 0.65 | < 0.001 | 0.88 |
| **DLEM** | 0.50 | < 0.001 | 0.52 |
| GTEC | 0.60 | < 0.001 | 4.25 |
| **ISAM** | 0.83 | < 0.001 | 0.15 |
| LPJ-wsl | 0.61 | < 0.001 | 1.82 |
| ORCHIDEE-LSCE | 0.59 | < 0.001 | 0.57 |
| SIBCASA | 0.65 | < 0.001 | 0.58 |
| VEGAS2.1 | 0.60 | < 0.001 | 0.25 |
| **Climate** | | | |
| **CLM4** | 0.05 | 0.7 | 0.08 |
| **CLM4VIC** | -0.15 | 0.26 | -0.15 |
| **DLEM** | 0.005 | 0.98 | 1.07 |
| GTEC | -0.41 | 0.002 | -8.74 |
| **ISAM** | -0.4 | 0.003 | -25.64 |
| LPJ-wsl | 0.06 | 0.67 | 0.11 |
| ORCHIDEE-LSCE | 0.15 | 0.28 | 6.97 |
| SIBCASA | -0.31 | 0.02 | -5.91 |
| VEGAS2.1 | -0.54 | < 0.001 | -1.39 |
| FLUXNET-MTE | -0.08 | 0.6 | -6.937× 10^-5^ |

Table S2. Summary of statistical analysis of models CUE for 1901-2010. % change is calculated based on Sen’s slope and mean CUE (See supplementary information). Mann-Kendall is estimated for simulation differencing except for BG1. Thus, only models with submitted simulations RG1 through BG1 (models with coupled C-N cycle) and RG1 through SG3 (Carbon cycle only models) are included. Models with coupled carbon-nitrogen cycle are denoted in bold.

|  | **Mann-Kendall** | **p-value** | **% change** |
| --- | --- | --- | --- |
| **BG1** | | | |
| **CLM4** | -0.12 | 0.06 | -0.42 |
| **CLM4VIC** | -0.04 | 0.56 | -0.18 |
| **DLEM** | -0.39 | < 0.001 | -1.5 |
| **ISAM** | 0.52 | < 0.001 | 5.18 |
| **TEM6** | 0.64 | < 0.001 | 9.13 |
| **N-deposition** | | | |
| **CLM4** | 0.73 | < 0.001 | 0.66 |
| **CLM4VIC** | 0.71 | < 0.001 | 0.79 |
| **DLEM** | -0.53 | < 0.001 | -0.15 |
| **ISAM** | 0.48 | < 0.001 | 0.007 |
| **TEM6** | 0.69 | < 0.001 | 0.44 |
| **CO2** | | | |
| **CLM4** | -0.63 | < 0.001 | -3.11 |
| **CLM4VIC** | -0.49 | < 0.001 | -3.08 |
| **DLEM** | -0.48 | < 0.001 | -0.43 |
| GTEC | 0.47 | < 0.001 | 2.04 |
| **ISAM** | 0.39 | < 0.001 | 1.21 |
| LPJ-wsl | 0.46 | < 0.001 | 2.03 |
| ORCHIDEE-LSCE | 0.44 | < 0.001 | 0.22 |
| SIBCASA | 0.42 | < 0.001 | 2.08 |
| **TEM6** | 0.74 | < 0.001 | 1.71 |
| VEGAS2.1 | -0.67 | < 0.001 | 0.002 |
| **Climate** | | | |
| **CLM4** | -0.11 | 0.09 | -0.06 |
| **CLM4VIC** | 0.02 | 0.98 | 0.13 |
| **DLEM** | -0.27 | < 0.001 | -2.14 |
| GTEC | -0.21 | < 0.001 | -7.19 |
| **ISAM** | -0.19 | 0.004 | -3.81 |
| LPJ-wsl | 0.01 | 0.85 | 0.14 |
| ORCHIDEE-LSCE | -0.13 | 0.04 | -0.32 |
| SIBCASA | -0.29 | < 0.001 | -1.44 |
| **TEM6** | -0.27 | < 0.001 | -14.91 |
| VEGAS2.1 | 0.02 | 0.8 | 0.09 |

Table S3. Simple linear regression adjusted R^2^ between annual CUE and annual GPP, annual NPP, and annual autotrophic respiration (R_a_) for the BG1 simulation for years 1901 to 2010.

| **Model** | **CUE vs. GPP** | **CUE vs. NPP** | **CUE vs. R_a_** |
| --- | --- | --- | --- |
| CLM4 | 0.08 | NS^1^ | 0.14 |
| CLM4VIC | 0.06 | NS | 0.11 |
| DLEM | 0.19 | 0.09 | 0.32 |
| ISAM | 0.25 | 0.59 | NS |
| TEM6 | 0.71 | 0.86 | 0.27 |

^1^ NS: not significant relationship (p-value > 0.05)

Table S4 Percentage increase in WUE due to CO_2_ fertilization (See Supplementary Information). Models with coupled C-N cycle are indicated in bold

| Model | % WUE |
| --- | --- |
| CLM4 | 10 |
| CLM4VIC | 10 |
| DLEM | 11 |
| GTEC | 17 |
| ISAM | 9 |
| LPJ-wsl | 9 |
| ORCHIDEE-LSCE | 21 |
| SiBCASA | 23 |
| VEGAS2.1 | 1 |

Table S5. Percentage change in GPP, NPP, R_a_, and evapotranspiration (ET) for environmental drivers: Climate and CO_2_ fertilization. Models with coupled C-N cycle are indicated in bold

|  | **%GPP** | **%NPP** | **%Ra** | **%ET** |
| --- | --- | --- | --- | --- |
| **Climate** | | | |  |
| **CLM4** | 7 | 2 | 7 | 5 |
| **CLM4VIC** | 6 | 3 | 6 | 5 |
| **DLEM** | 0.4 | 0.4 | 0.7 | 5 |
| GTEC | 0.1 | 0.2 | 0.2 | 6 |
| **ISAM** | 0.7 | -0.2 | 3 | 4 |
| LPJ-wsl | 8 | 3 | 7 | 6 |
| ORCHIDEE_LSCE | 4 | 1 | 4 | 7 |
| SiBCASA | 1 | 0 | 2 | 5 |
| **TEM6** | 9 | 5 | 11 | - |
| VEGAS2 | 5 | 1 | 5 | 12 |
| **CO_2_** | | | |  |
| **CLM4** | 14 | 13 | 14 | 3 |
| **CLM4VIC** | 14 | 14 | 13 | 4 |
| **DLEM** | 15 | 14 | 16 | 4 |
| GTEC | 16 | 17 | 18 | 0 |
| **ISAM** | 9 | 12 | 7 | 1 |
| LPJ-wsl | 16 | 23 | 10 | 5 |
| ORCHIDEE_LSCE | 26 | 32 | 20 | 5 |
| SiBCASA | 27 | 32 | 24 | 3 |
| **TEM6** | 17 | 22 | 10 | - |
| VEGAS2 | -14 | -17 | 2.5 | 9 |

Table S6. Percentage contribution of each of the environmental drivers to the BG1 WUE and CUE for C-N models (highlighted in bold) and SG3 WUE and CUE for C only models. % change is estimated for simulation differencing (See Supplementary Information). Thus, only models with submitted simulations RG1 through BG1 (models with coupled C-N cycle) and RG1 through SG3 (Carbon cycle only models) are included.

|  | **% Climate** | **% CO2** | **% N dep** |
| --- | --- | --- | --- |
| **WUE** | | | |
| **CLM4** | 0.58 | 3.45 | 2.3 |
| **CLM4VIC** | 0.65 | 4.55 | 2.6 |
| **DLEM** | 0.79 | 5.56 | 2.4 |
| GTEC | -0.07 | 8.78 |  |
| **ISAM** | -0.69 | 8.97 | 0.01 |
| LPJ-wsl | 1.35 | 8.1 |  |
| ORCHIDEE-LSCE | -0.15 | 9.45 |  |
| SiBCASA | 0.41 | 10.25 |  |
| VEGAS2.1 | 5.86 | 2.7 |  |
| **CUE** | | | |
| **CLM4** | -0.05 | -0.18 | 0.53 |
| **CLM4VIC** | 0.08 | -0.18 | 0.53 |
| **DLEM** | -0.56 | -0.37 | -0.06 |
| GTEC | -0.47 | 1.86 |  |
| **ISAM** | 0.71 | 0.71 | 0.006 |
| LPJ-wsl | 0.44 | 1.33 |  |
| ORCHIDEE-LSCE | -0.01 | 0.42 |  |
| SiBCASA | -0.13 | 2.00 |  |
| **TEM6** | 0.42 | 1.04 | 0.42 |
| VEGAS2.1 | 4 | -1.2 |  |

Table S7. List of MsTMIP simulations required for the global 0.5^o^ ⨯ 0.5^o^ runs.

| Simulation name | Climate | Land cover | Atmospheric CO_2_ | Nitrogen deposition |
| --- | --- | --- | --- | --- |
| RG1 | constant | constant | constant | constant |
| SG1 | Time-varying | constant | constant | constant |
| SG2 | Time-varying | Time-varying | constant | constant |
| SG3 | Time-varying | Time-varying | Time-varying | constant |
| BG1 | Time-varying | Time-varying | Time-varying | Time-varying |

Table S8. MsTMIP models used to calculate CUE and WUE. See table S7 for information about MsTMIP simulations.

| MsTMIP Models | RG1 | SG1 | SG2 | SG3 | BG1 | Reference(s) |
| --- | --- | --- | --- | --- | --- | --- |
| CLM4 | **√** | **√** | **√** | **√** | **√** | Shi et al., 2011; Mao et al., 2012 |
| CLM4VIC | **√** | **√** | **√** | **√** | **√** | Li et al., 2011 |
| DLEM | **√** | **√** | **√** | **√** | **√** | Tian et al., 2011;  Tian et al., 2012 |
| GTEC | **√** | **√** | **√** | **√** |  | Post et al., 1997 |
| ISAM | **√** | **√** | **√** | **√** | **√** | Jain et al., 2009 |
| LPJ-wsl | **√** | **√** | **√** | **√** |  | Sitch et al., 2003 |
| ORCHIDEE-LSCE | **√** | **√** | **√** | **√** |  | Krimmer et al., 2005 |
| SiBCASA | **√** | **√** | **√** | **√** |  | Schaefer et al., 2008, 2009 |
| TEM6 | **√** | **√** | **√** | **√** | **√** | Hayes et al., 2011 |
| VEGAS2.1 | **√** | **√** | **√** | **√** |  | Zeng et al., 2005 |

**Supplementary Figures**
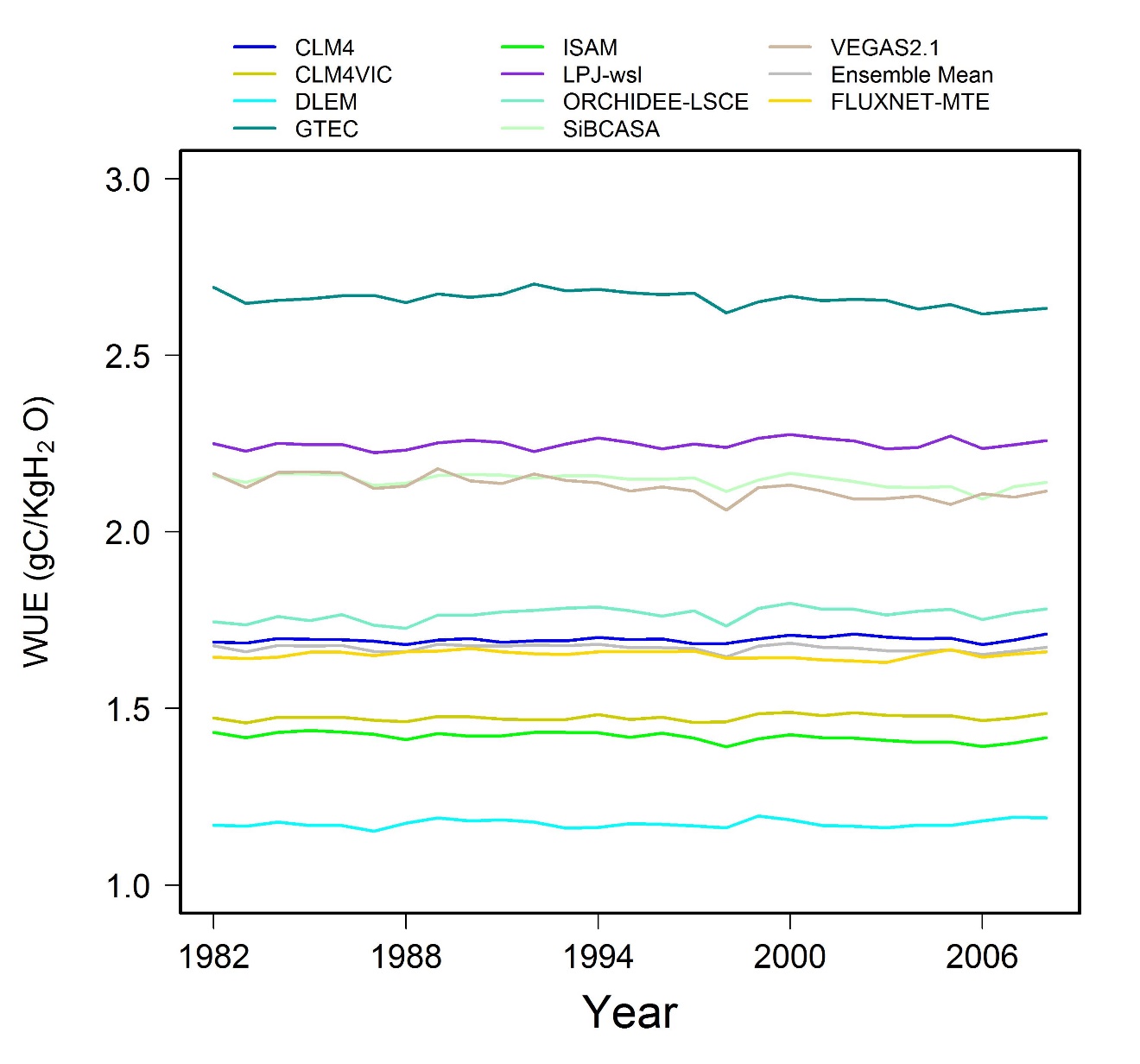


Figure S1. Climate change effect on models WUE. Time series plot showing the annual variability in WUE for the 11 models for the SG1 simulation and FLUXNET-MTE WUE for the time period of 1982-2008.


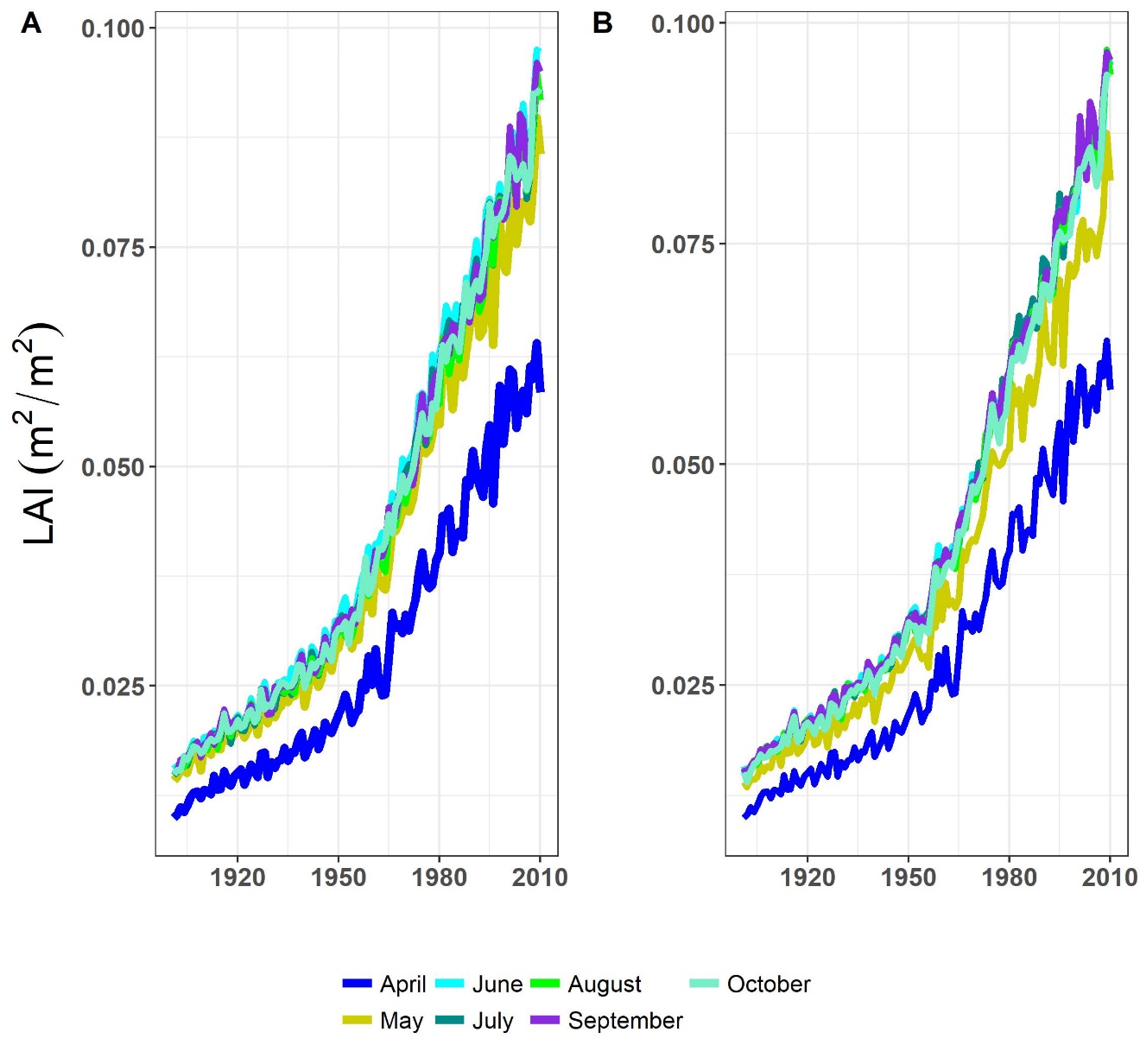


Figure S2. Long-term changes in LAI. Annual change in LAI due to N deposition effect (BG1-SG3) for (a) CLM4 and (b) CLM4VIC for years 1901-2010 (See methods).


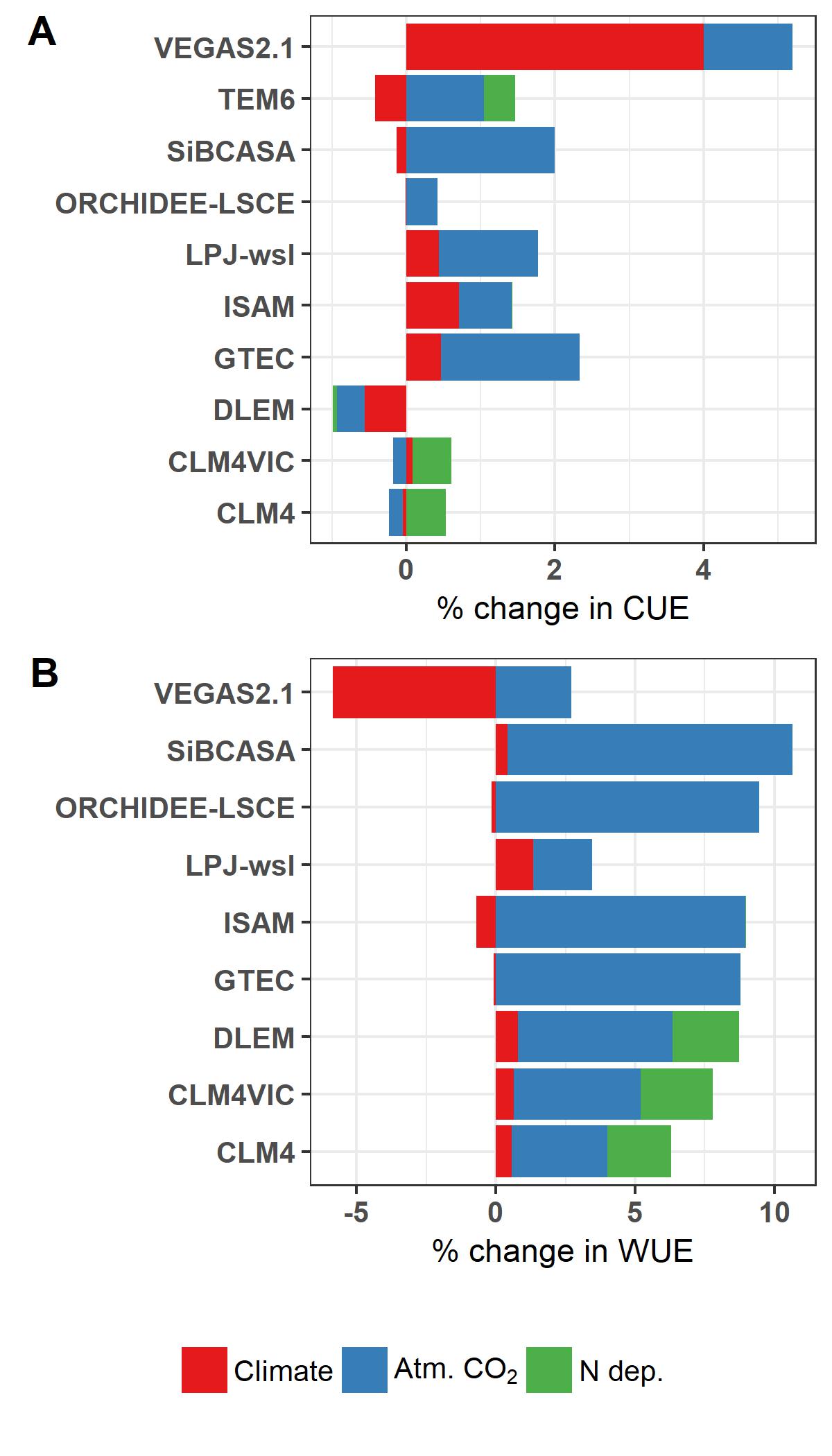


Figure S3. Attribution of environmental drivers (Climate, CO_2_ fertilization and N deposition) to percentage change on A: CUE; and B: WUE (See methods).


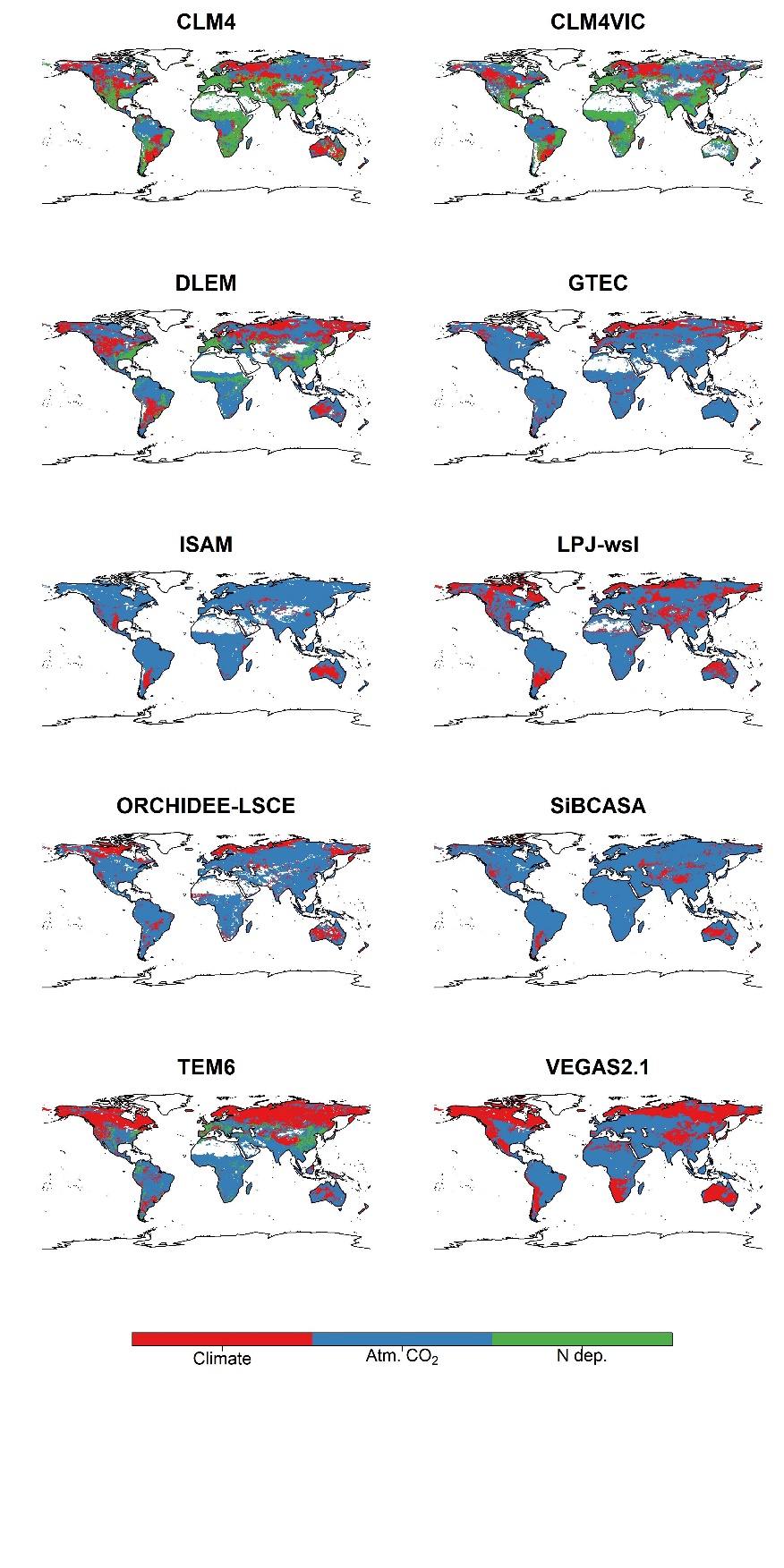


Figure S4. Spatial variability in the dominant environmental driver (Climate, CO2 fertilization and N deposition) on the average models GPP for 1982-2008. The averaged modeled GPP is for BG1 scenario (C-N models: CLM4, CLM4VIC, DLEM, and ISAM) and SG3 scenario (C-only models).


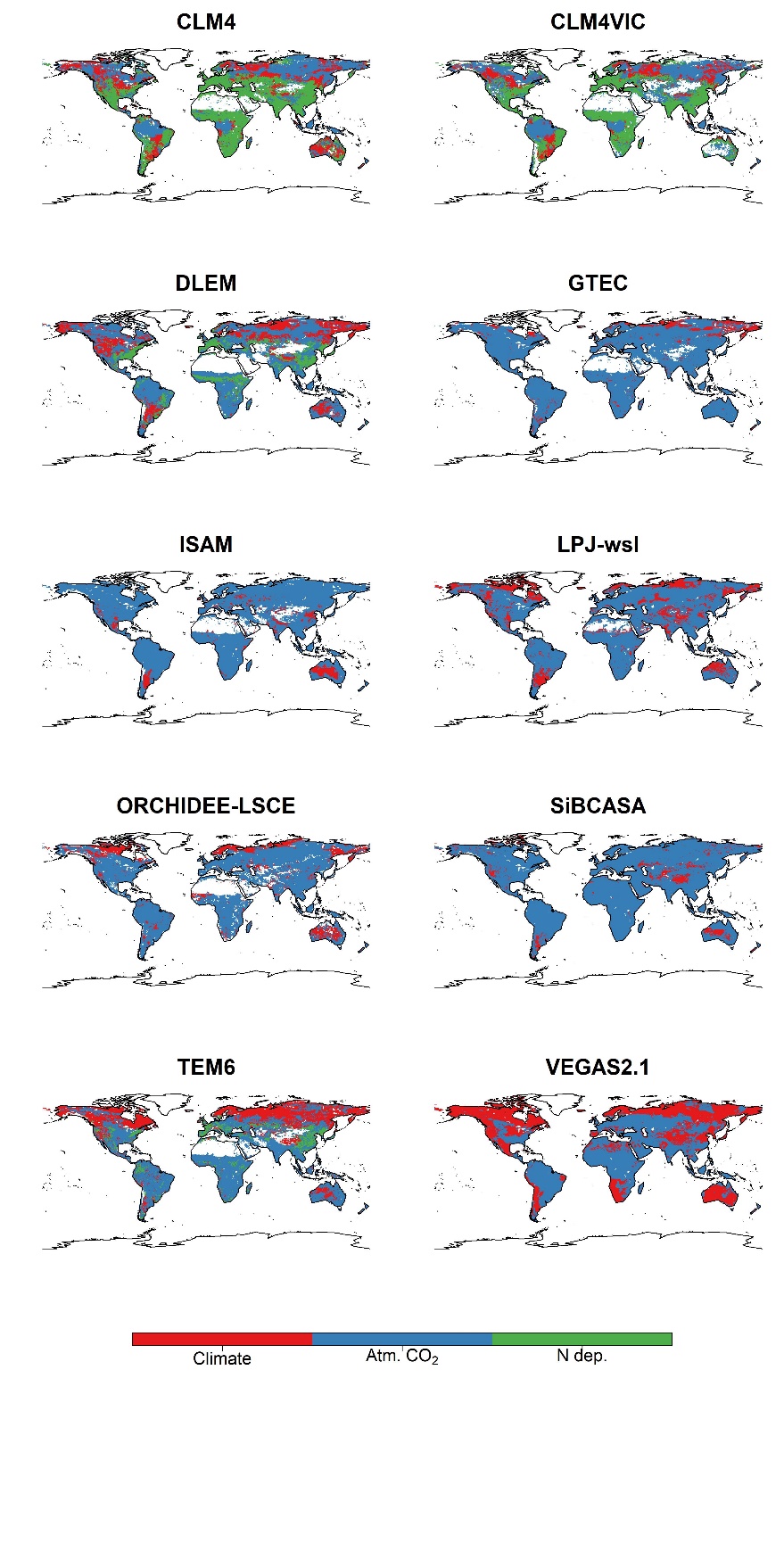


Figure S5. Spatial variability in the dominant environmental driver (Climate, CO2 fertilization and N deposition) on the average models NPP for 1982-2008. The averaged modeled NPP is for BG1 scenario (C-N models: CLM4, CLM4VIC, DLEM, and ISAM) and SG3 scenario (C-only models).


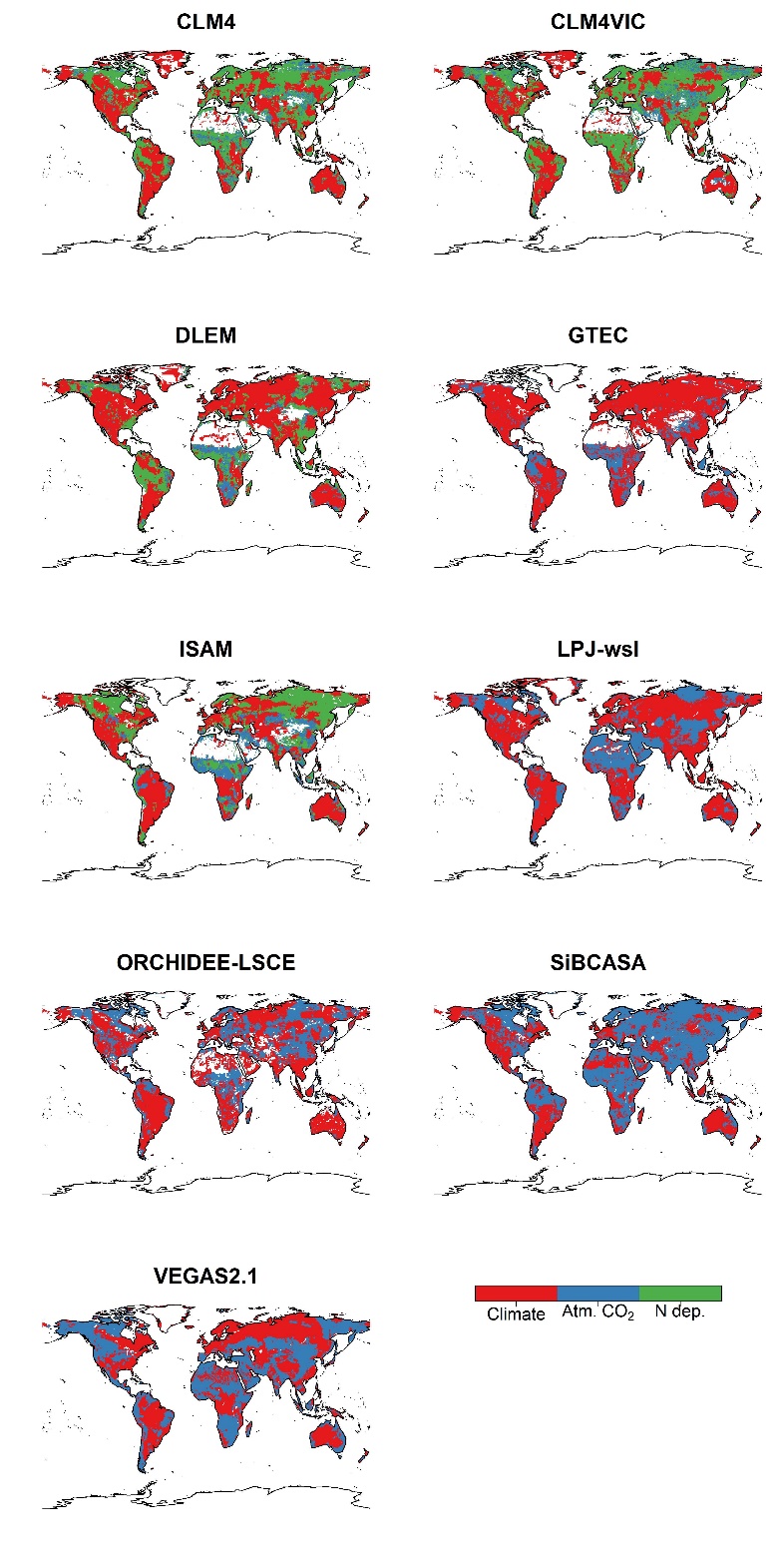


Figure S6. Spatial variability in the dominant environmental driver (Climate, CO2 fertilization and N deposition) on the average models ET for 1982-2008. The averaged modeled ET is for BG1 scenario (C-N models: CLM4, CLM4VIC, DLEM, and ISAM) and SG3 scenario (C-only models).


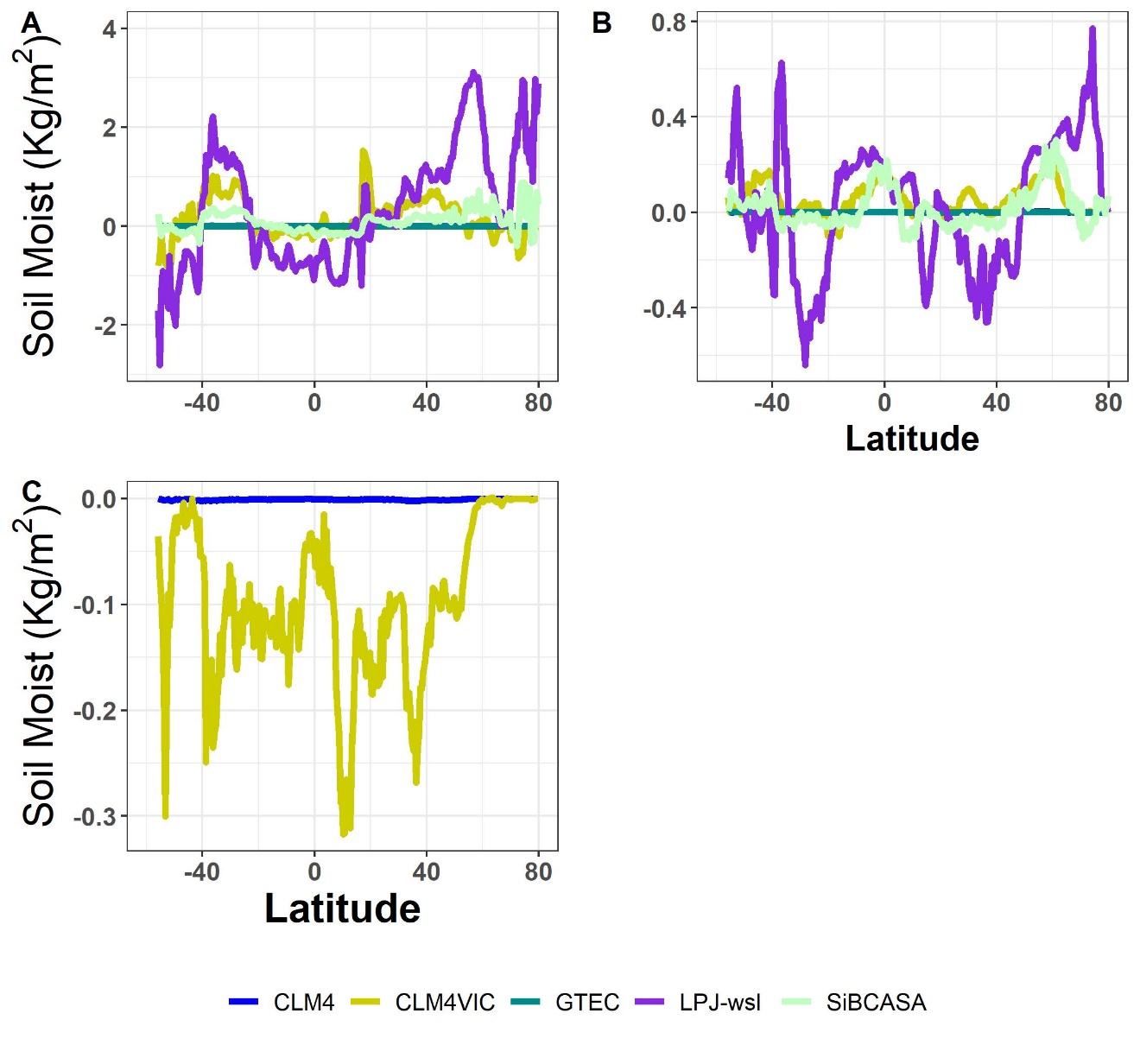


Figure S7. Latitudinal variability on models mean soil moisture for 1982-2008 due to (a) climate effect on models soil moisture calculated as the difference between SG1-RG1 simulations.; b) CO_2_ fertilization effect on models soil moisture calculated as the difference between SG3-SG2, (c) N deposition effect on models soil moisture calculated as the difference between BG1-SG3. Soil moisture is calculated as the average soil moisture for the top 6 soil layers, except for LPJ-wsl as only one soil moisture layer is used.


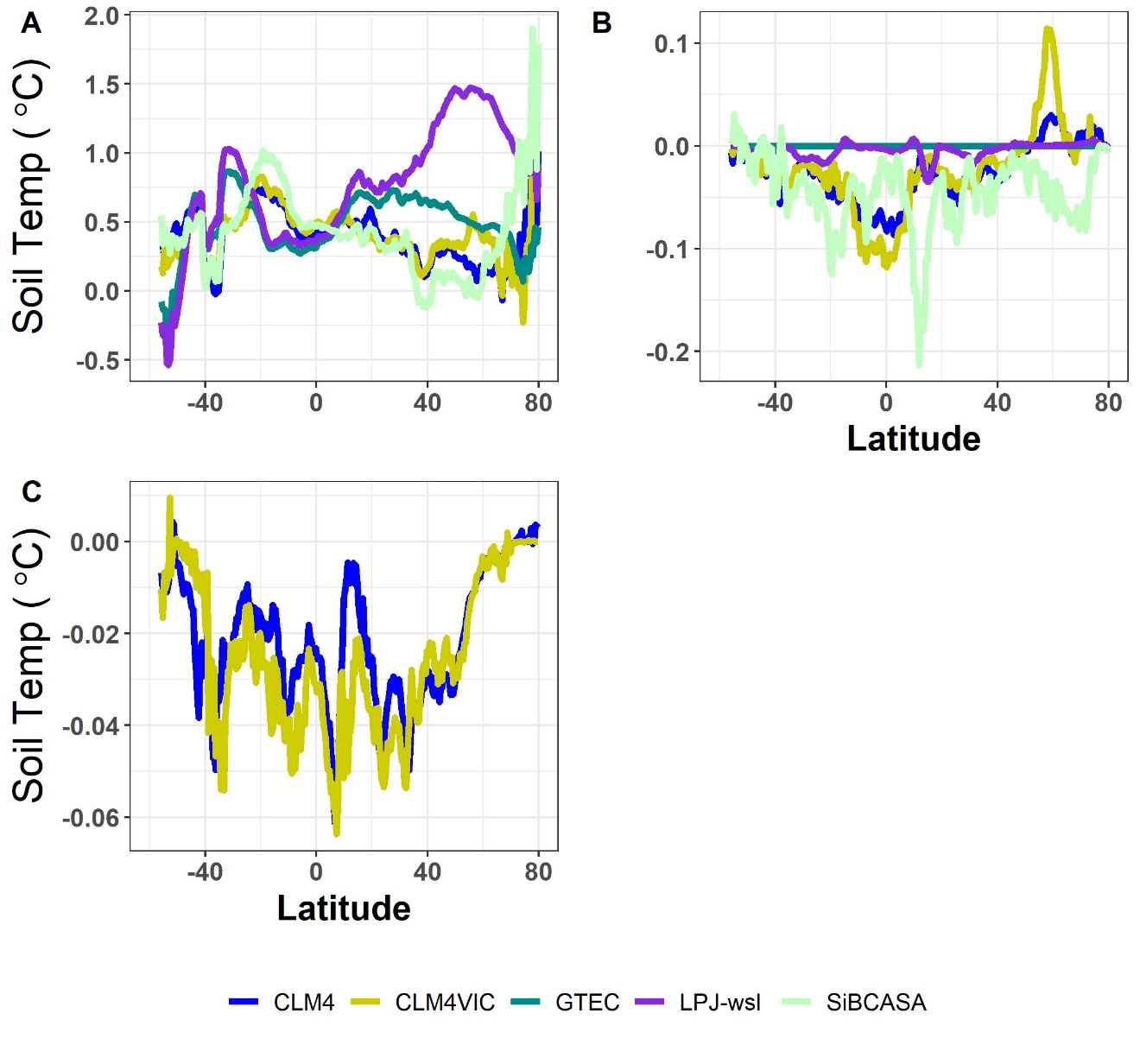


Figure S8. Latitudinal variability on models mean soil temperature for 1982-2008 due to (a) climate effect on models soil temperature calculated as the difference between SG1-RG1 simulations.; b) CO_2_ fertilization effect on models soil temperature calculated as the difference between SG3-SG2, (c) N deposition effect on models soil temperature calculated as the difference between BG1-SG3. Soil temperature is calculated as the average soil temperature for the top 6 soil layers for CLM4, CLM4VIC, and SiBCASA. GTEC and LPJ provide the average soil temperature for all soil layers.


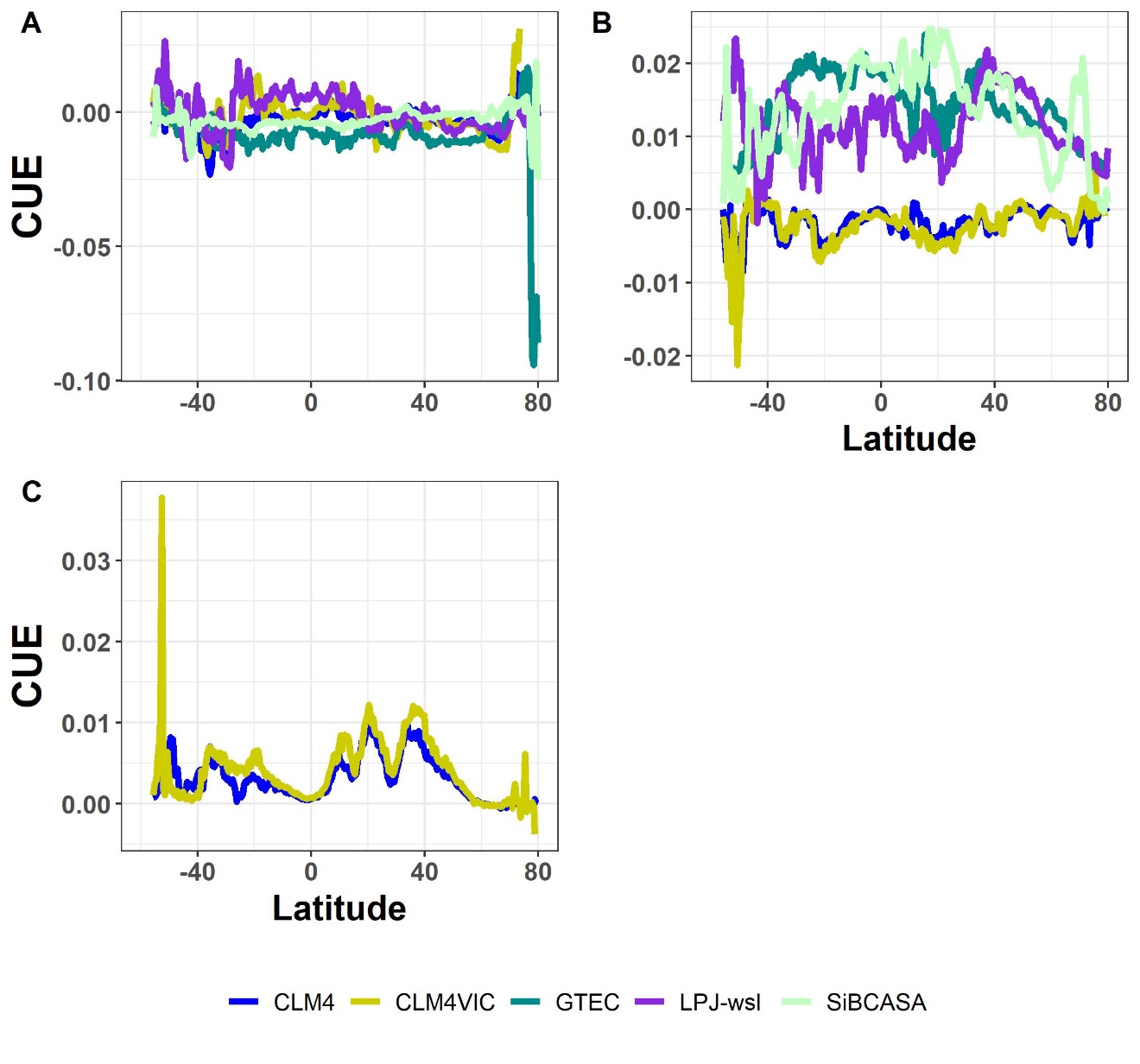


Figure S9. Latitudinal variability on models mean CUE for 1982-2008 due to (a) climate effect on models CUE calculated as the difference between SG1-RG1 simulations.; b) CO_2_ fertilization effect on models CUE calculated as the difference between SG3-SG2, (c) N deposition effect on models CUE calculated as the difference between BG1-SG3.


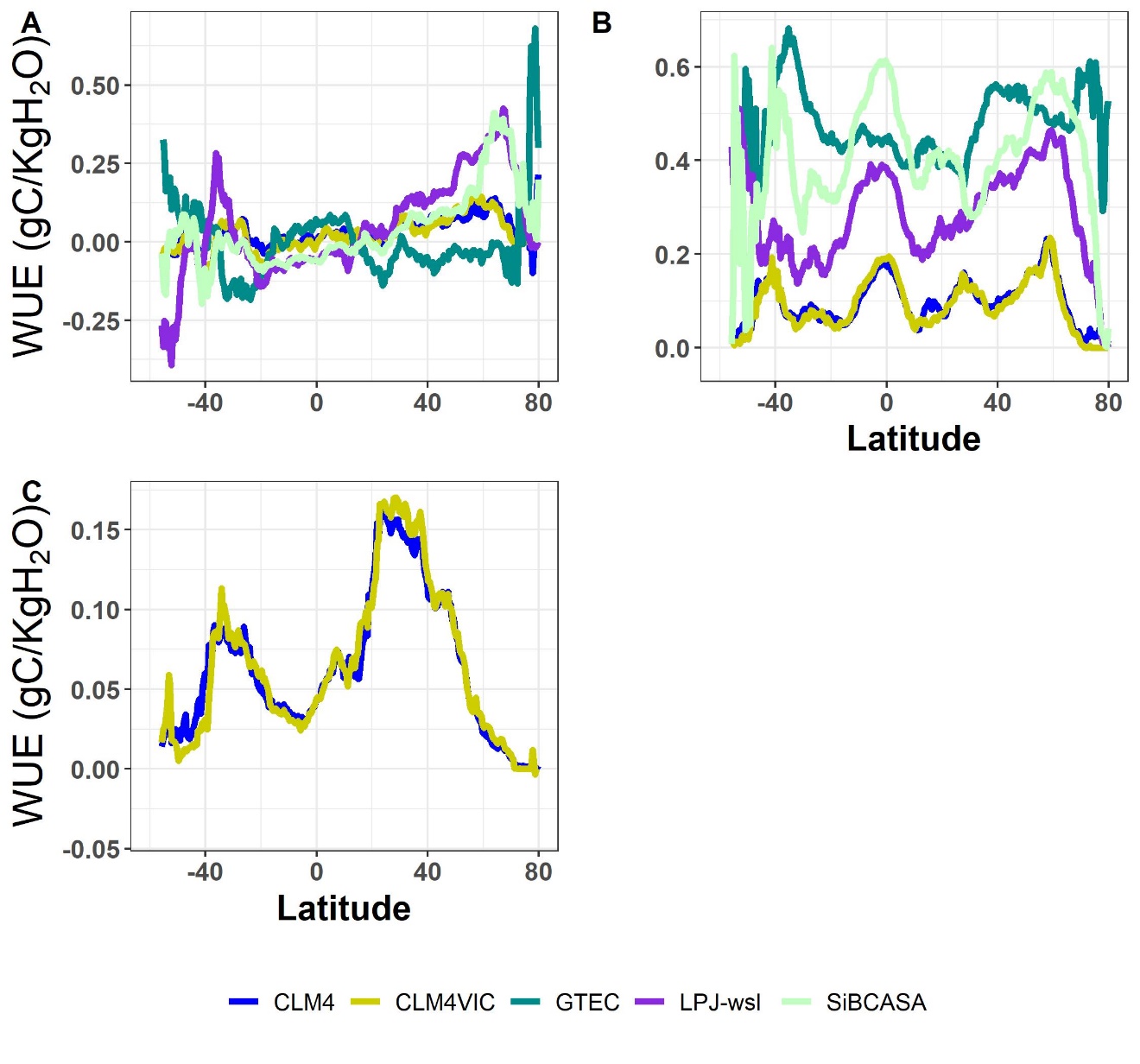


Figure S10. Latitudinal variability on models mean WUE for 1982-2008 due to (a) climate effect on models WUE calculated as the difference between SG1-RG1 simulations.; b) CO_2_ fertilization effect on models WUE calculated as the difference between SG3-SG2, (c) N deposition effect on models WUE calculated as the difference between BG1-SG3.

**References**:

1. Shi, X. Y., Mao, J. F., Thornton, P. E., Hoffman, F. M. & Post, W. M. The impact of

climate, CO2, nitrogen deposition and land use change on simulated contemporary global river flow. *Geophys. Res. Lett.* **38**, L08704 (2011).

2. Mao, J. F., Thornton, P. E., Shi, X. Y., Zhao, M. S. & Post, W. M. Remote sensing evaluation of CLM4 GPP for the period 2000-09. *J. Climate* **25**, 5327-5342 (2012).

3. Li, H. Y. *et al.* Evaluating runoff simulations from the Community Land Model 4.0

using observations from flux towers and a mountainous watershed. *J. Geophys. Res.*

*Atmos.* **116**, D24120 (2011).

3. Tian, H. Q. *et al.* Net exchanges of CO2, CH4, and N2O between China's terrestrial ecosystems and the atmosphere and their contributions to global climate warming. *J.Geophys. Res. Biogeosci.* **116**, G02011 (2011).

4. Tian, H. Q. *et al.* Century-scale responses of ecosystem carbon storage and flux to multiple environmental changes in the southern United States. *Ecosystems* **15**, 674-694 (2012).

5. Post, W. M., King, A. W. & Wullschleger, S. D. Historical variations in terrestrial biospheric carbon storage. *Glob. Biogeochem. Cycles* **11**, 99-109 (1997).

6. Jain, A. *et al.* Nitrogen attenuation of terrestrial carbon cycle response to global environmental factors. *Glob. Biogeochem. Cycles* **23**, GB4028 (2009).

7. Sitch, S. *et al.* Evaluation of ecosystem dynamics, plant geography and terrestrial carbon cycling in the LPJ dynamic global vegetation model. *Glob. Change Biol.* **9**, 161-185 (2003).

8. Krinner, G. *et al.* A dynamic global vegetation model for studies of the coupled atmosphere-biosphere system. *Glob. Biogeochem. Cycles* **19**, GB1015 (2005).

9. Schaefer, K. *et al.* Combined Simple Biosphere/Carnegie-Ames-Stanford Approach terrestrial carbon cycle model. *J. Geophys. Res. Biogeosci.* **113**, G03034 (2008).

10. Schaefer, K. *et al.* Improving simulated soil temperatures and soil freeze/thaw at high-latitude regions in the Simple Biosphere/Carnegie-Ames-Stanford Approach model. *J. Geophys. Res. Earth Surf.* **114**, F02021 (2009).

11. Hayes, D. J. *et al.* Is the northern h 328 igh-latitude land-based CO2 sink weakening? *Glob. Biogeochem. Cycles* **25**, GB3018 (2011).

12. Zeng, N., Mariotti, A. & Wetzel, P. Terrestrial mechanisms of interannual CO2 variability. *Glob. Biogeochem. Cycles* **19**, GB1016 (2005).
